# High accuracy gender determination using the egg shape index

**DOI:** 10.1038/s41598-023-27772-4

**Published:** 2023-01-10

**Authors:** Muhammed Kayadan, Yunus Uzun

**Affiliations:** Department of Electrical and Electronics Engineering, Aksaray University, Aksaray, Turkey

**Keywords:** Electrical and electronic engineering, Computational science

## Abstract

Since only female chicks are used in layer hens, usually hatched male chicks are killed. It is estimated that around 7 billion chicks per year are killed immediately after hatching. In addition to being unethical, this situation also causes great financial losses. Sex determination in chicks can be done before or after hatching. Of course, determinations made before hatching are more advantageous, but the prediction rate is relatively low. The morphology of an egg is expressed in terms of the Shape Index (SI), which is the ratio of the short diameter to the long diameter. In this study, male and female chicks were predicted by using the shape index of the eggs using the RUSBoost Classifier using Shape Index. Although SI varied according to the egg type, a significant correlation (r = 0.78) was observed between chick sex and SI. Therefore, it was possible to estimate gender by utilizing SI in chickens, even if the accuracy of classification was not as high as in ducks. Besides the SI, mass, short axis, long axis, ovality, volume, eccentricity parameters were obtained and used for the results. With this features, females classified with 80% and males classified 81% correctly. The model predictions were applied to the probability of female chick hatching equation from the previous studies, 71% of the estimations were correctly classified according to this equation.With this work, around 80% of accurate predictions were made. In this case, killing 5.65 billion chicks can be prevented. Likewise, many eggs are not wasted. 1.13 billion USD loss can be prevented.

## Introduction

Feeding and breeding activities are carried out to provide the necessary nutrients to the rapidly growing world population. The egg sector is one of the main sectors in the world. To increase egg production, breeding studies are carried out and studies on the chickens are continuing. Due to efficiency reasons, broiler and layer species have been selected for breeding by different features. Broilers grow fast and become ready to cut in short time. In the other hand, layers do not grow that fast and will not be heavy when they develop. Male and female numbers of chicken eggs are close to each other^1^. The presence of male chicks in the egg sector is a major problem. They do not make eggs, nor do they make a profit when they are grown and sold for slaughter. Therefore, male chicks are selected by the sexers and are culled unethically using different methods such as slaughter, gas strangulation, or strangulation in an oxygen-free environment when they have just hatched. A minority are painted and sold in pet shops^2^.

Assuming that the male chicks are not separated, they will consume a lot of feed up to slaughter and the income received after slaughter will be much less than the expense. A rooster eats an average of one hundred grams of feed per day. In a farm which has 100 K chickens, in case the roosters are not separated, 100 K roosters will take place in the farm. Only the daily feed mass of the roosters will be 10 K kg. Considering that roosters eat a feed with a price of 1 USD per kilogram, 10 K USD per day means that only the feed money will go for the roosters. On the other hand, the kilogram price of their meat will be a maximum of 2 USD and it will be 2 kg when they develop. If there is no waste at the end of the 6-month average feed consumption, 2 USD * 2 kg * 100 K Pieces = 400 K USD income will be obtained. In contrast, 10 K USD * 6 Months * 30 Days = 1800 K USD expense. In this case, there is a loss of 1.400 K USD.

The number of birds per unit area is decisive on their health and profitability^3^. There should be 6–7 chickens per square meter^4^. Approximately 15,500 m^2^ of extra space is required for 100 K roosters. If it is calculated that 100 K roosters hatch from all eggs without wastage, 100 K eggs are hatched. Assuming an average egg is 0.2 USD, it means a loss of 20 K USD because of egg expenses. 7 billion male chicks are culled annually in the world^2^. This is a loss of 1.4 billion USD. In addition to all these, economic losses will occur in many items such as the use of extra incubators, extra electrical energy spent for the incubator, and the number of personnel to work.

In the hatchery, the sexers separate chicks into male and female. In the egg sector, because only the female chickens are useful, it is necessary to separate them. Chick sex can be determined in three different stages. These are sex determination before incubation, during incubation, and after incubation. In this work, sex determination before incubation is used because this is the most useful method to save the life of the chick inside or hatched eggs and to prevent the waste of eggs.

It is possible to determine the gender with great accuracy before the eggs are incubated, and the most determinant measure in this method is the egg shape index^5–10^. In^7^, 300 White Nick Super Layer eggs were examined with an unsupervised regression method, and female hatching probability was suggested. In^8^, 340 Pekin duck eggs were studied, and 0.71 positive correlation coefficient was found. In^9^, 103 duck eggs were examined and 86% accuracy was obtained. In^10^, 503 duck eggs were used with different machine learning algorithms and 87% accuracy was obtained. There are some different techniques, such as the use of fluorescence^11,12^, Raman spectroscopy^13^, egg shape index^8^, DNA glycosylase^14^, to detect sex of the chicks. Except for the morphological methods, all the methods need complicated, expensive tools and the eggs were perforated. In-ovo egg sexing methods are applicable only during the incubation, therefore male eggs are getting wasted. The shape index method can be applicable before incubation; thus male eggs can be sold to the market for daily consumption. Furthermore, the earliest period of in-ovo methods is the ninth day of the incubation; hence, sources will still be used, and alive-in-egg chicks will be culled. There is a need for an industry-applicable, reliable, fast-working method on chicken breeds. Because of these reasons, industrial in-ovo selection devices are still not fully applicable in the poultry industry. The studies which it used the shape index method are mostly on ducks, but duck egg models cannot be applied to chicken eggs because of their different morphology. Dikmen^7^ suggested a regression analysis, instead of a classification model. We are suggesting our classification model for chicken breeds, which does not harm eggs and is very fast.

**Figure 1:**
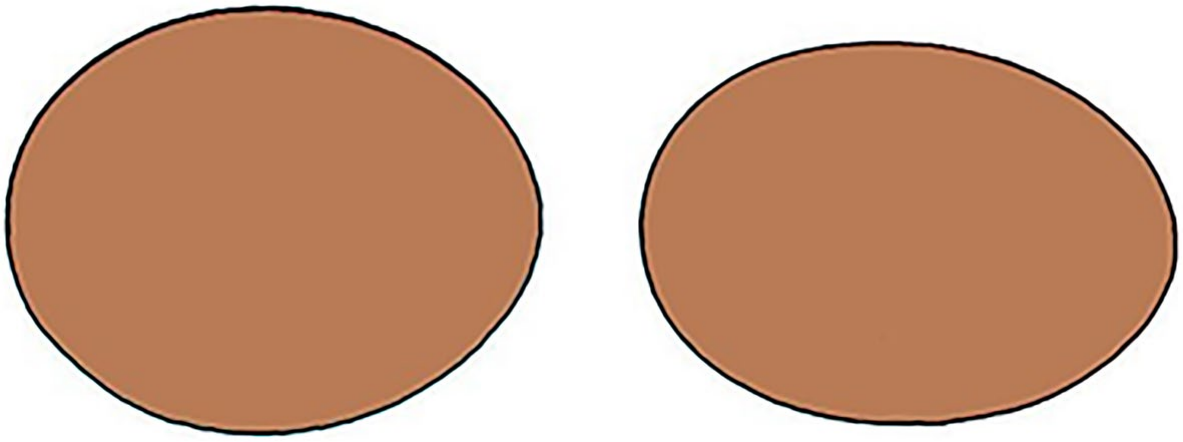
Oval shape egg for female chicks (left) and pointed shape egg for male chicks (right).

In this study, sex determination using the shape index of the egg is preferred since the other methods are both costly and difficult to use in serial analysis. The shape index (SI) is the ratio of the short diameter of the egg to the long diameter^15,16^. In general, eggs with a low shape index will be male chicks, eggs with a high shape index will be female chicks. In general, eggs are of two types, oval shape and pointed shape, as seen in Fig. 1.

The shape index (SI) is the ratio of the short diameter of the egg to the long diameter. When using the egg shape index, the standard deviation of the shape index is quite low. (0.03844 mm in 60 eggs) So sex discrimination is very difficult. Therefore, it was decided to use different methods in comparison. In this way, possible performance comparison was aimed. The results obtained will be compared with the sex of the hatching chicks and the method with the highest accuracy will be determined.

In accordance with all these conditions, the following methods have been decided:

- Sex classification with a supervised learning algorithm. RUSBoosted was used in this study.
- Determine the shape index by directly measuring the short and long axis of the egg, and gender discrimination according to the threshold value.

Specify both eccentricity (automatic SI) and short and long axis shape index (manual SI) using MATLAB Image Processing Toolboxwith this study, the relation between shape index of the eggs and sex of chicks was questioned, and the results were listed.

## Methodology

**Figure 2:**
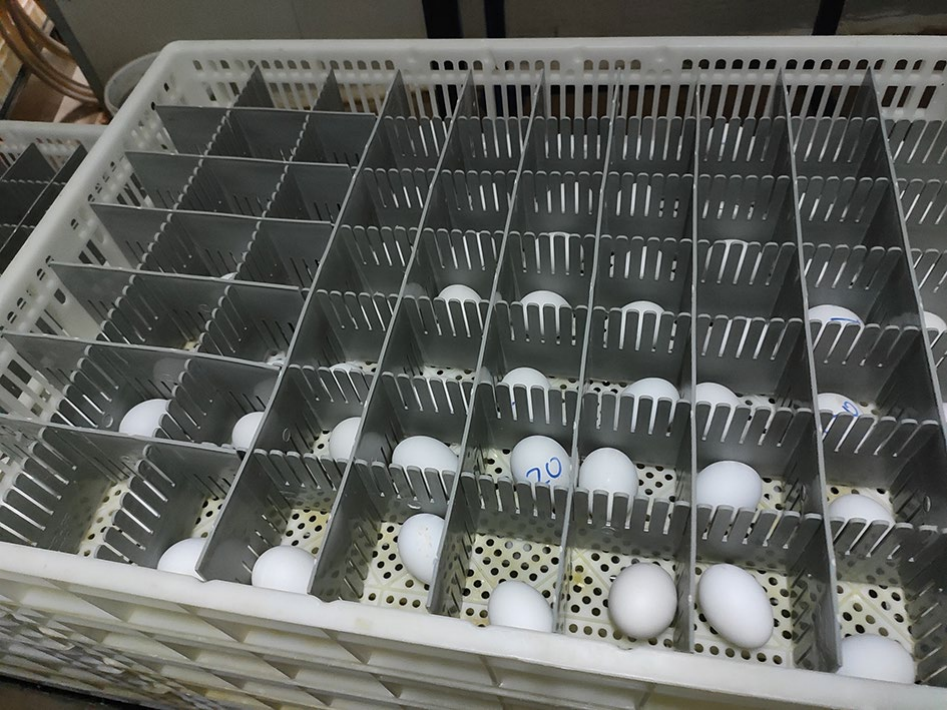
Divided incubation tray structure.

The experiment was carried out in the Aksaray University hatcheries in accordance with the national regulations on wellness and protection of animals used for experimental and other scientific purposes. Eggs for the experiment were bought from the local backyard poultry farms. The morphology of an egg varies on the chicken breed and in the local backyard farm had a hybrid flock. Egg images were taken before the incubation. Eggs were clamped down by their long axis and camber alignment tools was used to lay eggs down most accurately. (Mitutoyo 06,389,100, 0.01 mm accuracy) Each egg was given an identical number (from 0 to 60), and their ID number was written on eggs and their corresponding incubation tray cells with a marker. First, 18 days of incubation eggs were arranged in numerical order, however, the last 3 days’ incubator trays were divided into cells for tracking chicks. It is crucial for sex pairing between eggs and chicks in supervised learning. Figure 2 shows the cell structure in the incubation tray. For the tray dividing process, netted materials were obligated to use due to air circulating. After hatching, the chicks were one week old, distinguishing them was done by visual inspection based on their body size, comb color, and feathering.

**Figure 3:**
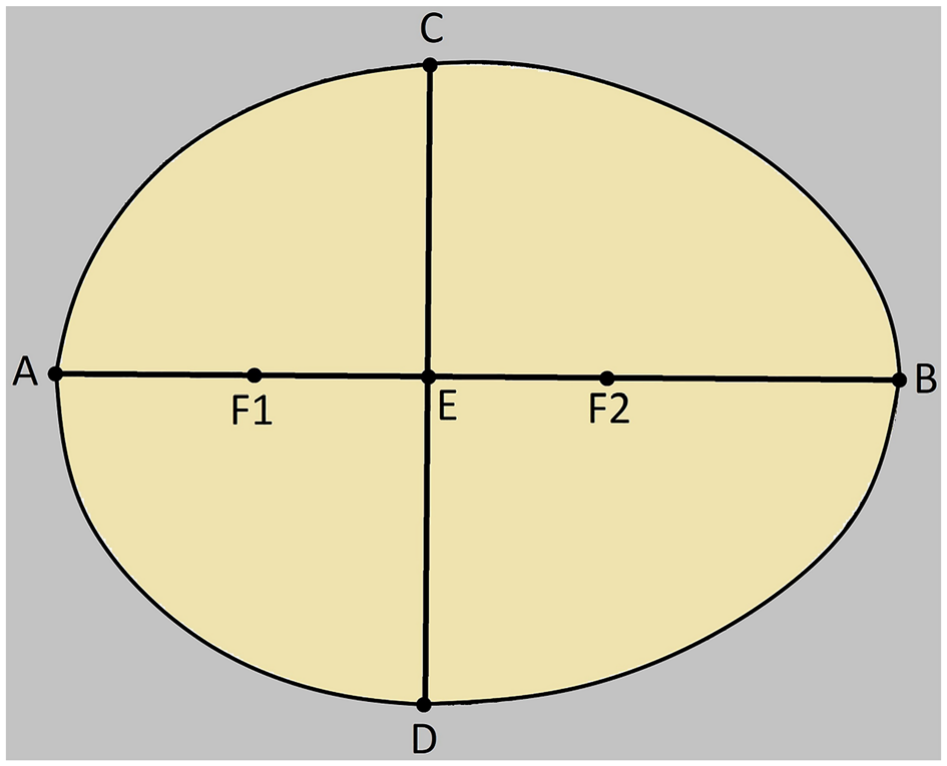
Alignment of an egg. Rounded part (**A**), Pointed part (**B**), Maximum width points (**C** and **D**), Focal Points (F1 and F2), Cross Point (**E**).

Mass is scaled with the Diheng THR219 0.01-g sensitive scale and recorded. For the physical measurements, short axis |CD|, long axis |AB|, and the maximum point |AE| (on where bumpy side (C point) touches to calliper) is measured with 0.01 mm sensitive digital calliper. In previous studies, Shape Index (SI) is used^7,8,16^. The Shape Index is the ratio of the short axis to the long axis and on Fig. 3:

1 $$Shape \,index = \frac{Short\, Axis}{Long\, Axis}=\frac{|CD|}{|AB|}$$

Shape Index can vary between 0 and 1. 0 refers to a line and 1 refers to a perfect circle. Ovality was created as a parameter in this study. |CD| line segment in Fig. 3 represents the maximum width of the egg. Ovality is described as

2 $$Ovality=\frac{Long\, Axis-Cross \,Point}{Long\, Axis}=\frac{|AB|-|AE|}{|AB|}$$

If the egg is a perfect ellipse, E point is placed in the middle of |AB| line and ovality becomes 0.5.

The image folder address was added to MATLAB. “*.jpg*” file extension was used for images.

In a loop up to the number of images, the photos were read (imread) and were resized to 540–720 for quick processing of reading images (imresize).

The3-channel RGB images were converted to single-channel gray images (rgb2gray). Single-channel gray images were converted to a black-and-white logical image (im2bw) with white below the threshold and black above. Since the threshold value of each image was not the same, a study was performed on the threshold value and 2 different threshold values were obtained. The first was the gray thresh and the second was the average of the gray image. The resulting black and white images were saved for analysis (imwrite).

Long axis, short axis, and eccentricity values of black and white images were obtained by using the function “*regionprops*”. The shape index was obtained from the short axis and long axis data (automatic shape index). The eccentricity is the ratio of the distance between the foci of the ellipse and its major axis length and can be shown in Eq.(3)as^16–18^:

3 $$Eccentricity=\frac{Distance\, between \,Fociis}{Long \,Axis}=\frac{|F1 F2|}{|AB|}$$

The value is between 0 and 1. (if the eccentricity of an ellipse, it is actually a circle, if the eccentricity of an ellipse is 1, it is a line segment^17,18^. Obtained shape index, eccentricity, short and long axis were saved in Excel file “.xlsl” for studying (xlswrite). After creating the Excel file and fundamental parameters, some additional parameters were created for further inspection. Surface area formula^19^:

4 $$Surface\, Area=4.835*{Mass}^{0.662}$$

Surface area was obtained using the above equation. The surface area and volume can be transformed from the geometry of the oval. Formula for obtaining volume from the surface area^19^:

5 $$Volume={\left(\frac{Surface \,Area}{4.951}\right)}^{\frac{1}{0.666}}$$

Obtained using the formula. Density from mass and volume information:

6 $$Density= \frac{Mass}{Volume}$$

Obtained with the formula. There is more than one formula for egg volume, but the formula that minimizes the standard deviation of egg density (0.000781369) was used. Probability of hatching female eggs^7^:

7 $$Probability \,of \,hatching\, female\, eggs = -0.39531+0.01214*SI*100$$

All measured values were saved on Microsoft Office Excel 2021 Professional and other calculations were performed on this platform. Mass (m), long axis (L), short axis (W), shape index (SI), probability of female chick growth, surface area (S), volume (V), density (d), ovality values were calculated for 60 eggs and minimum, maximum, average and standard deviation values of each parameter were calculated.

Since we knew the real sexes of the chicks, therefore eggs, supervised classification methods were used. We trained the RUSBoosted trees on MATLAB. RUSBoost trees algorithm is a classification technique for learning from disordered training data. This algorithm provides a simpler and faster alternative to SMOTEBoost, which is another algorithm that combines boosting and data sampling^20^. The correlation coefficient of all the variables was found with the Matlab corrcoef() built-in function. With this function coefficients matrix and *p* values matrix were obtained. The matrix of *p* values for testing the hypothesis that there is no relationship between the observed phenomena. General results and information are shown in Table 1.

**Table 1:** The results and information of the RUSBoosted-tree model on MATLAB.

Results	
Accuracy	80.9%
Prediction Speed	~ 310 obs/sec
Training Time	4.1265 s
Model Tune	
Preset	RUSBoosted Trees
Ensemble Method	RUSBoost
Learner Type	Decision Tree
Maximum Number of Splits	20
Number of Learners	30
Learning Rate	0.1
Feature Selection	
Before PCA, all features use except:	Sex of Chick
PCA	
PCA status	Disabled

Model features totalled 8 consists of mass, short axis, long axis, shape index from the calliper, shape index from MATLAB, eccentricity from MATLAB, and ovality. Both SI values because they do not completely overlap. Cross-validation was kept as 5 for preventing overfitting in our limited data. Principal Component Analysis (PCA) was kept closed. Features and their descriptions are shown in Table 2.

**Table 2:** Classification features and their descriptions.

Feature	Description
Mass (g)	The mass of the eggs. Mass is scaled with 1-g sensitive digital scale
Short axis (mm)	Minor axis of the egg. The short axis is measured with a 0.01 mm sensitive digital caliper. (|CD| on Fig. 3)
Long axis (mm)	Major axis of the egg. The long axis is measured with a 0.01 mm sensitive digital caliper. (|AB| on Fig. 3)
Foci (mm)	The distance between the rounded side of egg and where the rear side touches to the caliper. (|AE| on Fig. 3)
Shape index	Physical measured short and long axis ratio (|CD|/|AB|)
Automatic eccentricity	Eccentricity value which is taken from MATLAB regionprops
Automatic SI	Shape Index value which is the rate of MATLAB short and long axis
Ovality	The ratio between foci and long axis, measured physically. (|AE|/|AB)|

## Results and discussions

**Figure 4:**
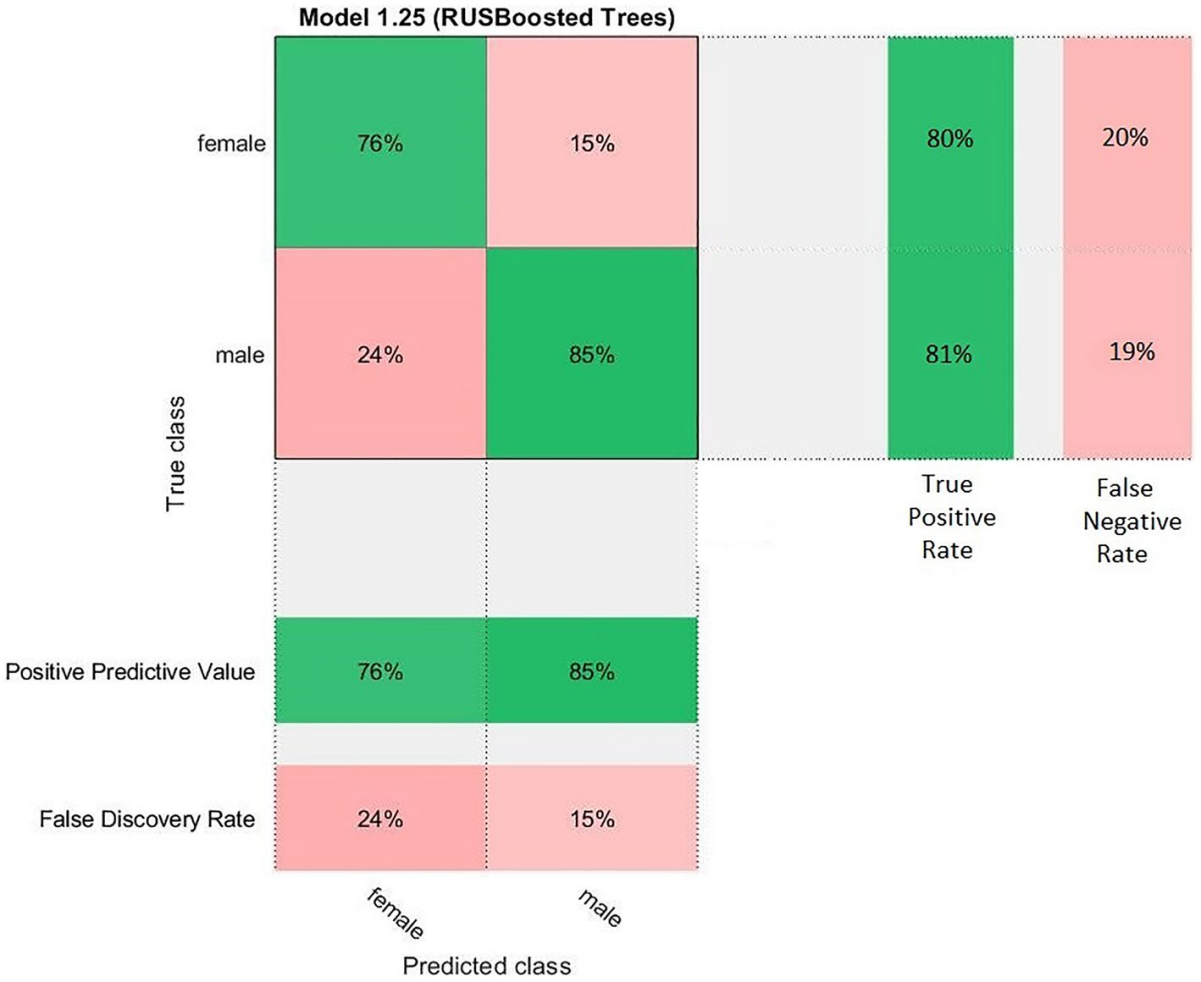
The Confusion Matrix for all classes. Rows represent the real sex of chicks which were determined after hatching, columns represent the classified sexes. Each quarter represents the corresponding accuracy. By quarters, up-left: 76% of the females were classified correct, bottom-left: 24% of the females were classified wrong, up-right: 15% of the males classified wrong, bottom-right: 85% of the males were classified correct.

The confusion matrix was created according to the results obtained from the software using the shape index. The confusion matrix displays the total number of observations in each cell. The rows of the confusion matrix correspond to the true class, and the columns correspond to the predicted class. Diagonal and off-diagonal cells correspond to correctly and incorrectly classified observations, respectively. The confusion matrix of our predicted data is shown in Fig. 4.

After the prediction, females classified 80% accurate and males classified 81% accurate. In the study on ducks^9^, it was found in our study that males have a higher hatching probability than females.

On the prediction side, if our model estimates an egg as a male, it will be 85% accurate, and if it predicts as female, it will be 76% accurate. It means that if our model one egg is male and the other is female, they are not equally trustable. A low shape index value means a very low probability of containing a female chick, but if we have a high shape index egg, it can contain a male egg with a higher chance comparatively.

At the end of the incubation period, forty-seven chicks were hatched out of 60 eggs. Eight out of the unhatched eggs were unfertilized and 5 of them were dead-in-shell as determined using candling. All the unhatched eggs were kept out of the evaluation. Chick sexing was done for alive forty-seven chicks. Female, male, and unhatched eggs are shown in Table 3.

**Table 3:** Egg and hatching numbers.

Number of eggs	60
Number of chicks	47
Number of male chicks	27
Number of female chicks	20
Number of unhatched eggs	13

**Figure 5:**
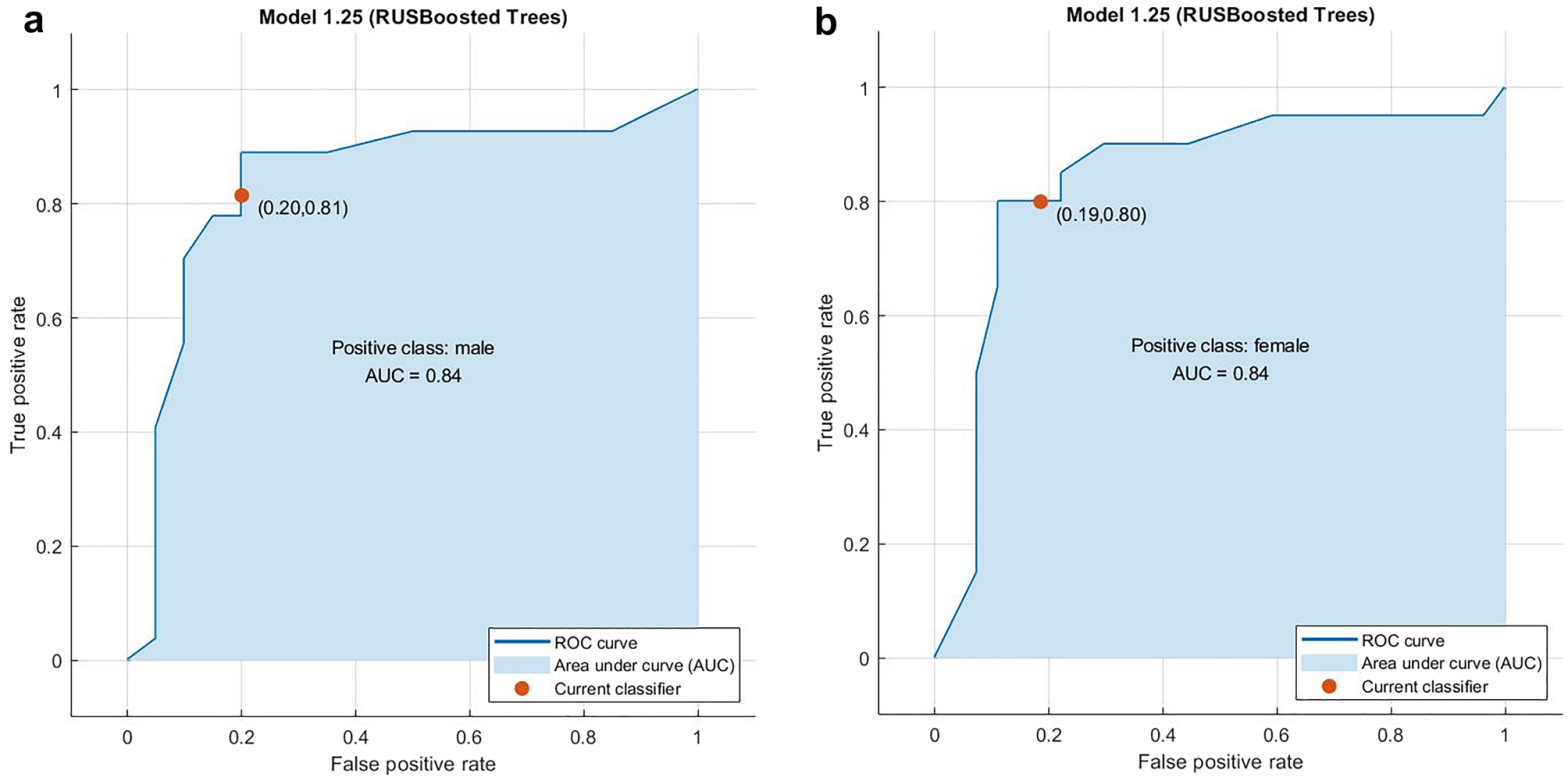
ROC analysis for male class (**a**) and female class (**b**).

The ROC curve shows the relationship between the true positive rate (TPR) for the model and the false positive rate (FPR). The TPR is the rate at which the classifier predicts “positive” for observations that are “positive.” The FPR is the rate at which the classifier predicts “positive” for observations that are actually “negative.” A perfect classifier will have a TPR of 1 and an FPR of 0. This analysis is mostly used for binary classifications. In our study, binary classes are male and female. ROC analysis graphic for males is shown in Figs. 5a and for females, it is shown in Fig. 5b. Area Under Curve (AUC) calculation has significant meaning in the graphics.

Parallel Coordinates Plots (PCP) are ideal for comparing many variables together and seeing the relationships between them. This type of visualization is used for plotting multivariate, numerical data.

**Figure 6:**
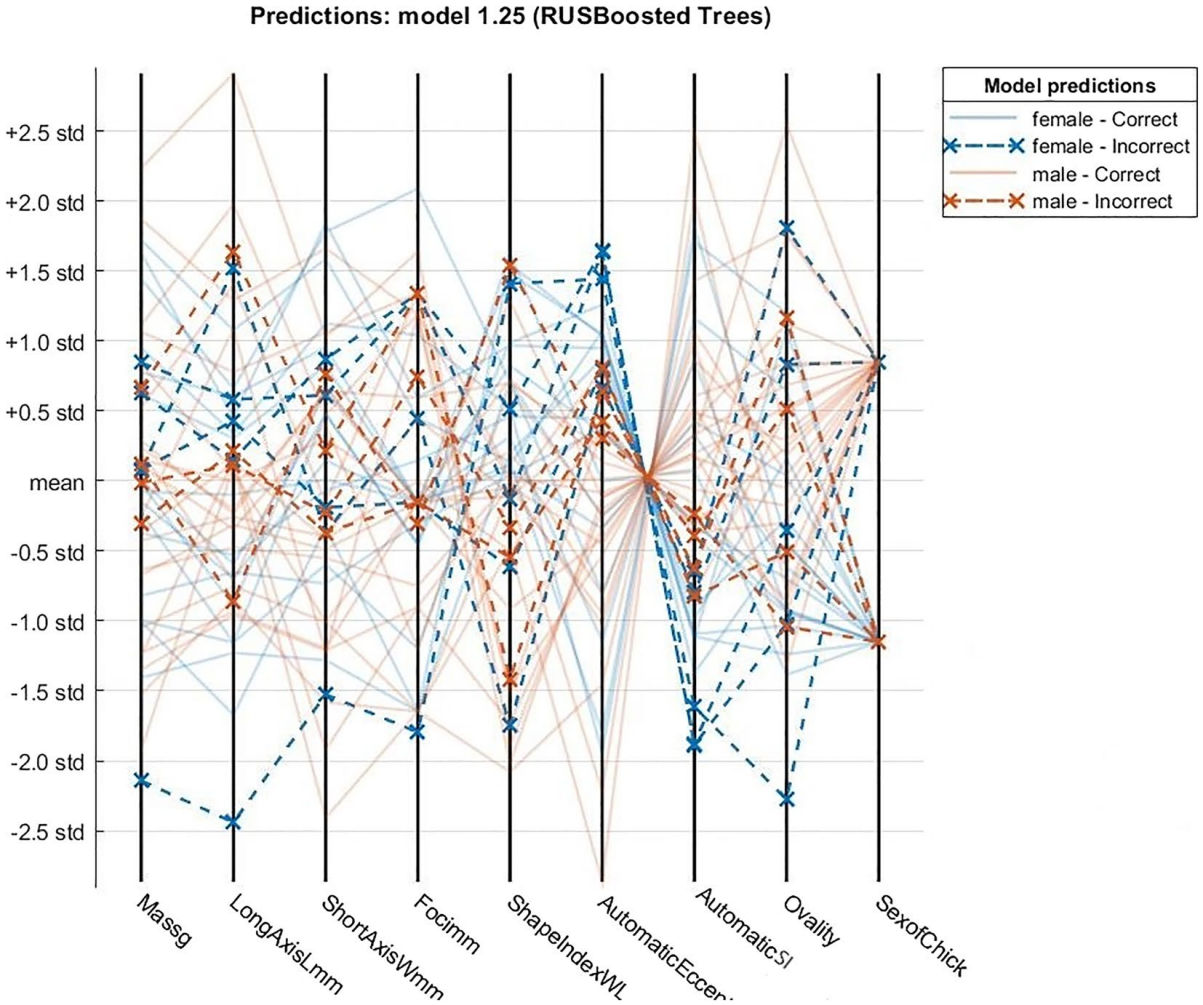
Parallel Coordinates Plot shows the relationship between variables. All features are normalized.

Figure 6 shows the PCP graphic for processed features and plots their distribution. Descriptions of features are shown in Table 2. As shown in the table mass and the long axis has the widest range. Crossing between Automatic Eccentricity and Automatic SI was expected, we know that low SI corresponds to high eccentricity by their definitions.

**Figure 7:**
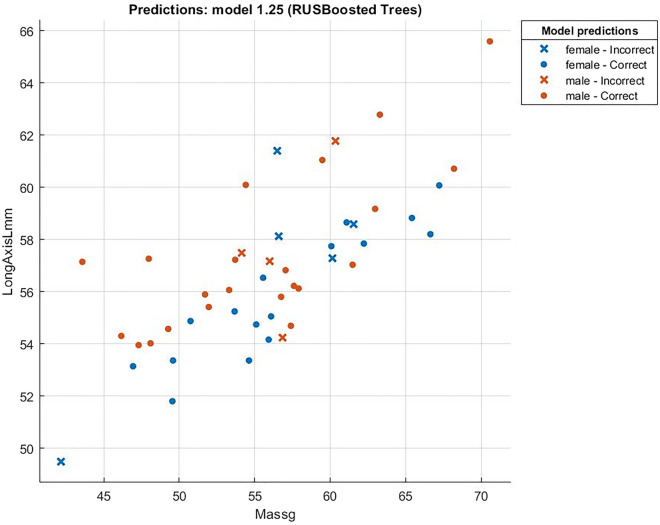
Correlation between mass and long axis (r = 0.76).

**Figure 8:**
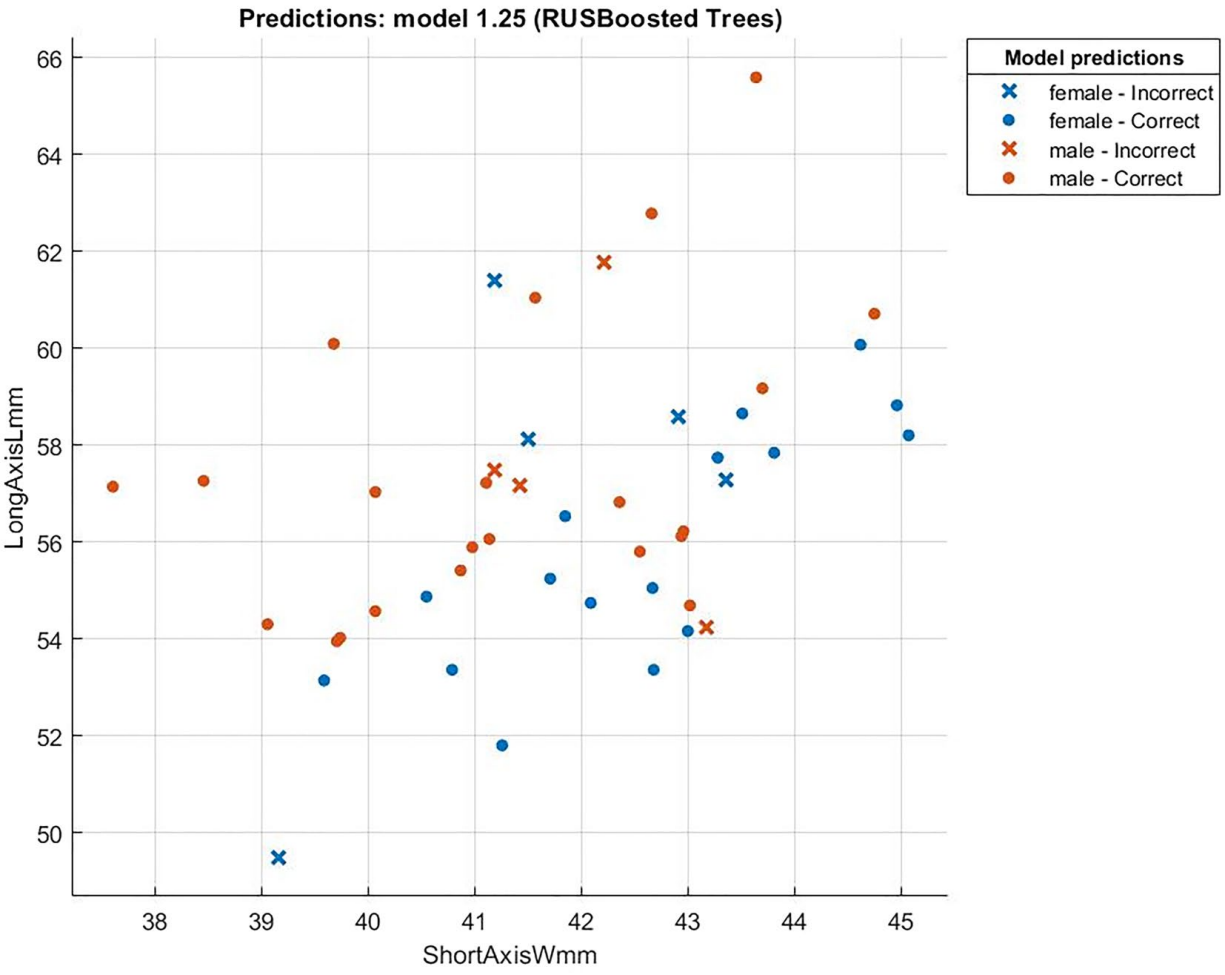
The relation between Short and Long Indexes. (r = 0.45).

Relation between mass and long axis is shown in Fig. 7, and it is shown that there is a positive correlation between parameters. This result was expected by the previous studies^7–9,15,16,19^. Cross points correspond to incorrect prediction. The eggs with longer axis values, thus bigger, were supposed to have higher mass. If an egg has the same mass, but a shorter long axis, it means that the short axis is higher, which means a higher shape index, and is predicted as more likely female. The relation between short and long indexes is shown in Fig. 8.

We can see some clusters in Fig. 8, it may cause by different chicken breeds. It is clear that there is a positive correlation, but the rate is very low.

## Conclusion

In this study, a total of 60 used eggs, and only 47 of them were hatched. This number is comparatively low, and a large number of data is needed for better results. Random eggs were chosen from local farms as breeds and the age of chickens, if a specific breed and age are to be used in future studies it may give better results. The shape of an egg may vary on the breed of chicken. During layer hen grows, the mass of egg increases, however, this growth is not homogeneous. As the hen ages, her egg enlarges towards the short axis, not the long axis, which changes the shape index directly.

The average of the estimations (0.5017) was considered as the threshold value. The estimation values below the threshold are considered male, above the threshold, are considered female. 37 out of 47 (0.787) chicks were classified correctly.

80.85% means that we can save 5.65 billion out of 7 billion killed male chicks. This, in addition to saving chick lives, means that the average profit of 20 cents^21^ is calculated as 1.13 billion USD per year.

By the deductive method, the image of a large number of eggs is taken and incubated. Egg images, which are numbered according to the sex values obtained from the incubation, are classified and given to the net for training and the net is trained. However, this method requires a large number of hatching eggs, sufficient staff for measurements, and a large incubator. In the study, it was determined that there was a high correlation between shape index and gender. In the next stage, it is planned to transform the design into a machine and separate the eggs which high probability of being female.

## Data Availability

Data are available upon request from the corresponding author.
